# Rickettsial infections of the central nervous system

**DOI:** 10.1371/journal.pntd.0007469

**Published:** 2019-08-29

**Authors:** Zuzana Sekeyová, Monika Danchenko, Peter Filipčík, Pierre Edouard Fournier

**Affiliations:** 1 Institute of Virology, Slovak Academy of Sciences, Dubravska cesta, Bratislava, Slovakia; 2 Institute of Neuroimmunology, Slovak Academy of Sciences, Dubravska cesta, Bratislava, Slovakia; 3 Aix-Marseille Univ, IRD, AP-HM, SSA, VITROME, IHU Mediterranée-Infection, Marseille, France; 4 Centre National de Référence des *Rickettsia*, *Coxiella* et *Bartonella*, IHU Mediterranée-Infection, Marseille, France; Faculty of Science, Ain Shams University (ASU), EGYPT

## Abstract

As a result of migrations and globalization, people may face a possible increase in the incidence of central nervous system rickettsial infections (CNS R). These diseases, caused by *Rickettsia* species and transmitted to humans by arthropod bites, are putatively lethal. However, the diagnosis of CNS R is challenging and often delayed due to their nonspecific clinical presentation and the strict intracellular nature of rickettsiae. Furthermore, transfer of rickettsiae to the brain parenchyma is not yet understood. The aim of this review is to analyze and summarize the features and correlated findings of CNS R in order to focus attention on these intriguing but frequently neglected illnesses. We also incorporated data on CNS infections caused by *Rickettsia-*related microorganisms.

## Introduction

Recent reports have warned about the impact of global warming in facilitating the transmission of certain vector-borne infectious diseases. However, due consideration must be taken of the role played by other variables, such as the increase in international travel, migration, and trade, with the risk of importing parasites and vectors with the goods [1]. Rickettsioses are common tick-, flea-, or mite-borne bacterial illnesses with a clinical spectrum ranging from a mild febrile illness to potentially life-threatening complications [2,3]. Rickettsial infections can affect many organs, including the central nervous system (CNS) [4]. The most common neurological manifestations reported in rickettsial infections include meningitis, encephalitis, and acute disseminated encephalomyelitis [5]. However, unilateral facial nerve palsy [6], cerebral infarction [7], or visual loss [8] have also been diagnosed. Therefore, it is critical to enhance the awareness of physicians worldwide on CNS rickettsial infections (CNS R).

Few studies on the pathogenesis of rickettsiae for the CNS have been conducted. In neurons cultured in vitro, rickettsiae have been demonstrated to cause a profound morphological deterioration and intracellular decrease of ATP [9]. In addition, it was demonstrated that rickettsiae can persist and reappear after a relatively long time in the CNS in immunocompromised mice, causing a fatal neuroinflammation [10]. This suggests a potential neuropathic role of rickettsiae. However, data on rickettsial infections in a context of degenerative CNS diseases, which usually result from mixed neuropathologies and present with multiple symptoms, are scarce in the literature, and to date, no association between neurodegenerative diseases and rickettsial infections has been demonstrated.

In this article, we systematically reviewed the literature reporting cases of CNS R to provide scientists and healthcare providers with a summary of the current knowledge on and challenges posed by these diseases. We also included data on *Orientia*, *Anaplasma*, and *Ehrlichia* species, which are phylogenetically close to *Rickettsia* species.

## Literature search and selection criteria

We searched PubMed with the following Medical Subject Heading terms: “rickettsia” (9,504 results); “rickettsia” AND “central nervous system” (169 results); and “rickettsia” AND “central nervous system” AND review (5 results). The search was limited to articles published in English between January 1, 1960, and February 28, 2019. In addition, no online book or book chapter in relation to this subject was identified.

We also searched PubMed with the Medical Subject Heading terms: “orientia” AND “human” (945 results); “ehrlichia” AND “human” (1,421 results); “anaplasma” AND “human” (1,385 results); “orientia” AND “human” AND “central nervous system” (26 results); “ehrlichia” AND “human” AND “central nervous system” (10 results); and “anaplasma” AND “human” AND “central nervous system” (3 results).

## Epidemiology of rickettsial diseases

**Fig 1:**
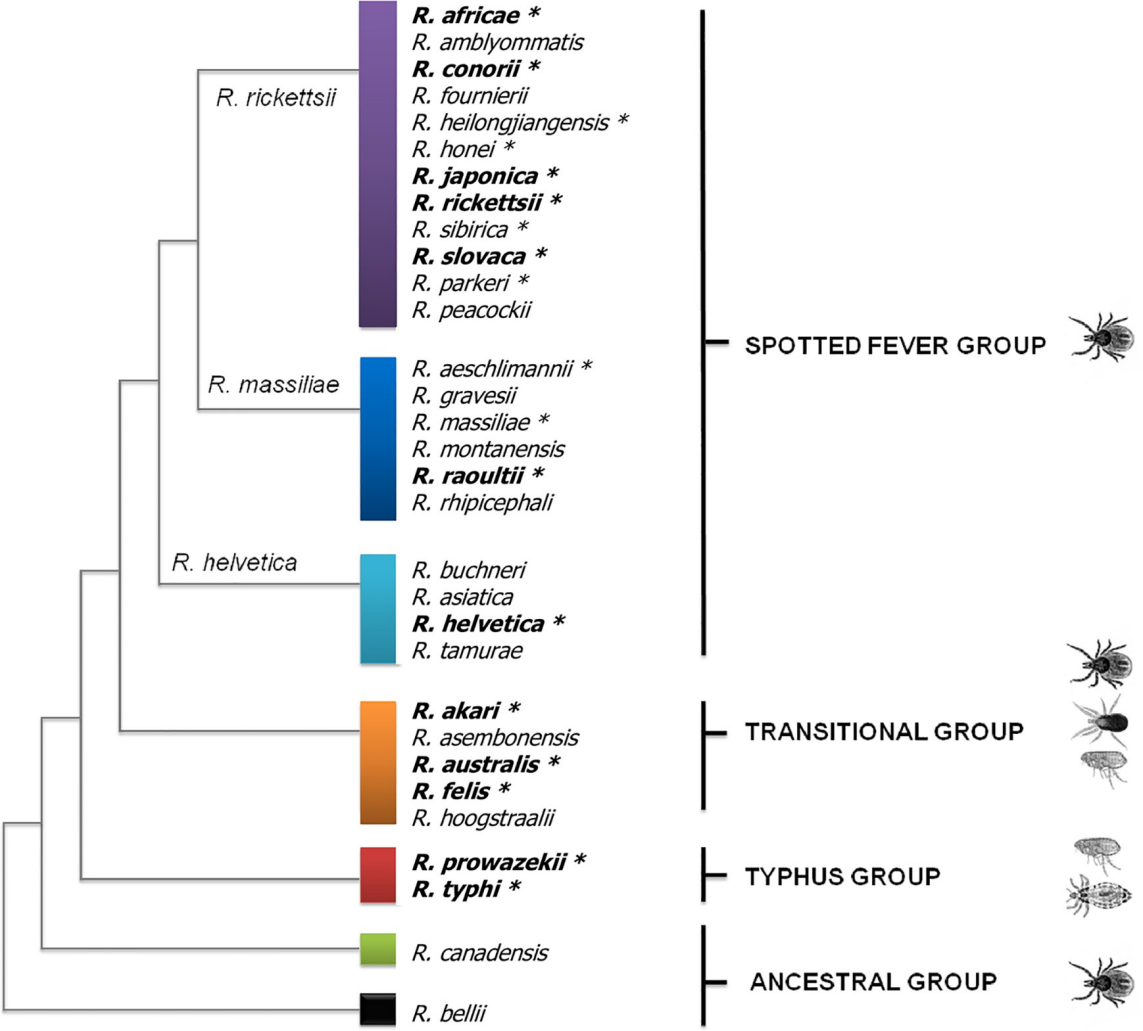
Phylogenetic tree of *Rickettsia* species inferred from the comparison of concatenated sequences from the *gltA* and *sca*4 genes. *Rickettsia* species are distributed into 4 groups: SFG, TRG, TG, and AG. SFG rickettsiae are mostly associated with ticks; TG rickettsiae with human body lice (*R*. *prowazekii*) and rat fleas (*R*. *typhi*); TRG rickettsiae with ticks, cat fleas, or mites; and AG rickettsiae with ticks. Asterisks mark pathogenic species, and bold letters indicate species causing CNS infections. AG, ancestral group; CNS, central nervous system; SFG, spotted fever group; TG, typhus group; TRG, transitional group.

**Fig 2:**
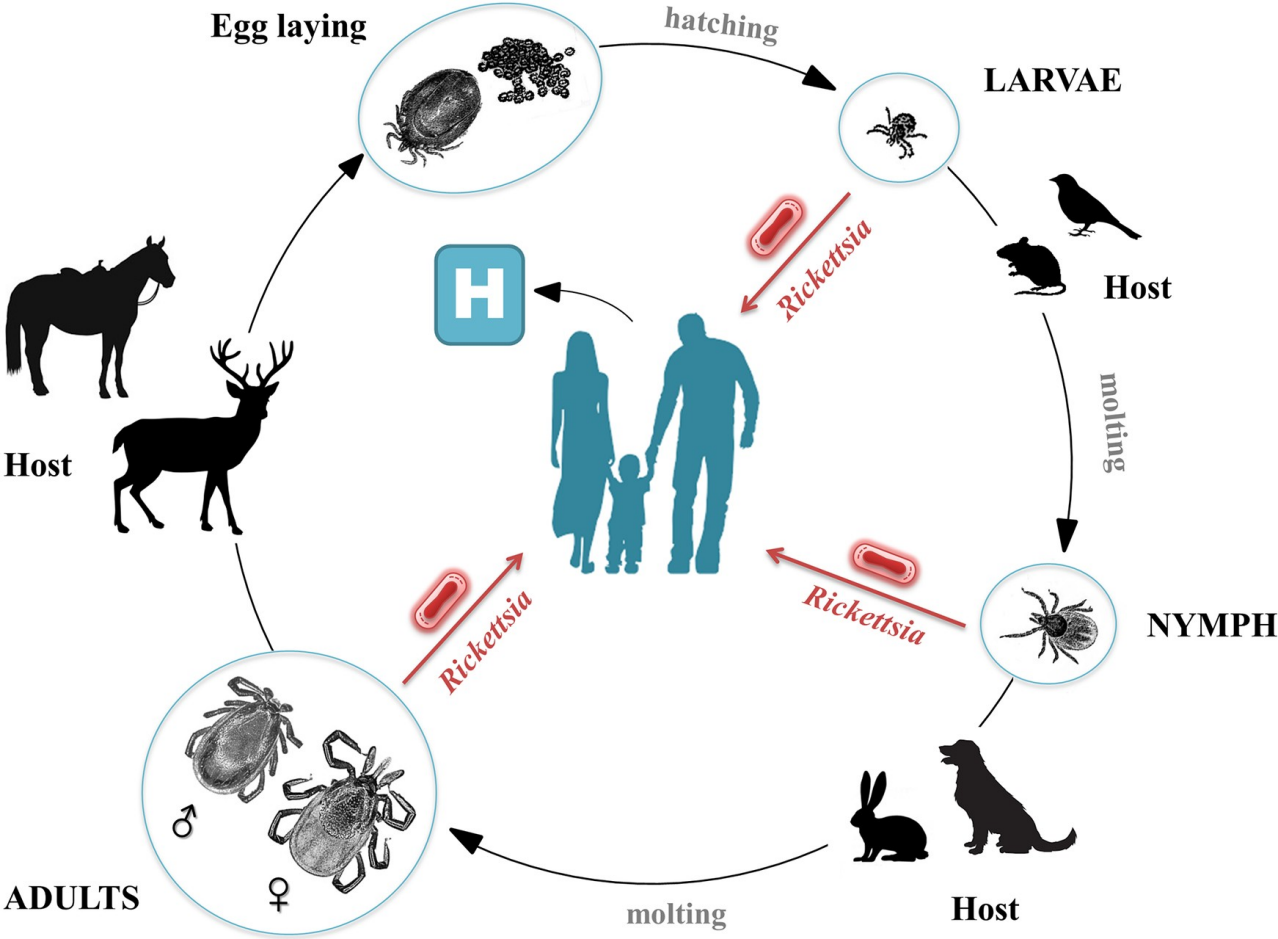
Developmental cycle and host range of *Rickettsia*-infected ticks. Ticks are the main vectors and constitute a threat of rickettsial infection regardless of their development stage (larvae, nymphs, or adults). Many factors play a role in the epidemiology of tick-borne rickettsioses, including the prevalence and species diversity of rickettsiae in mammals [149–153] and the dispersion of infected ticks by migratory birds [154].

**Fig 3:**
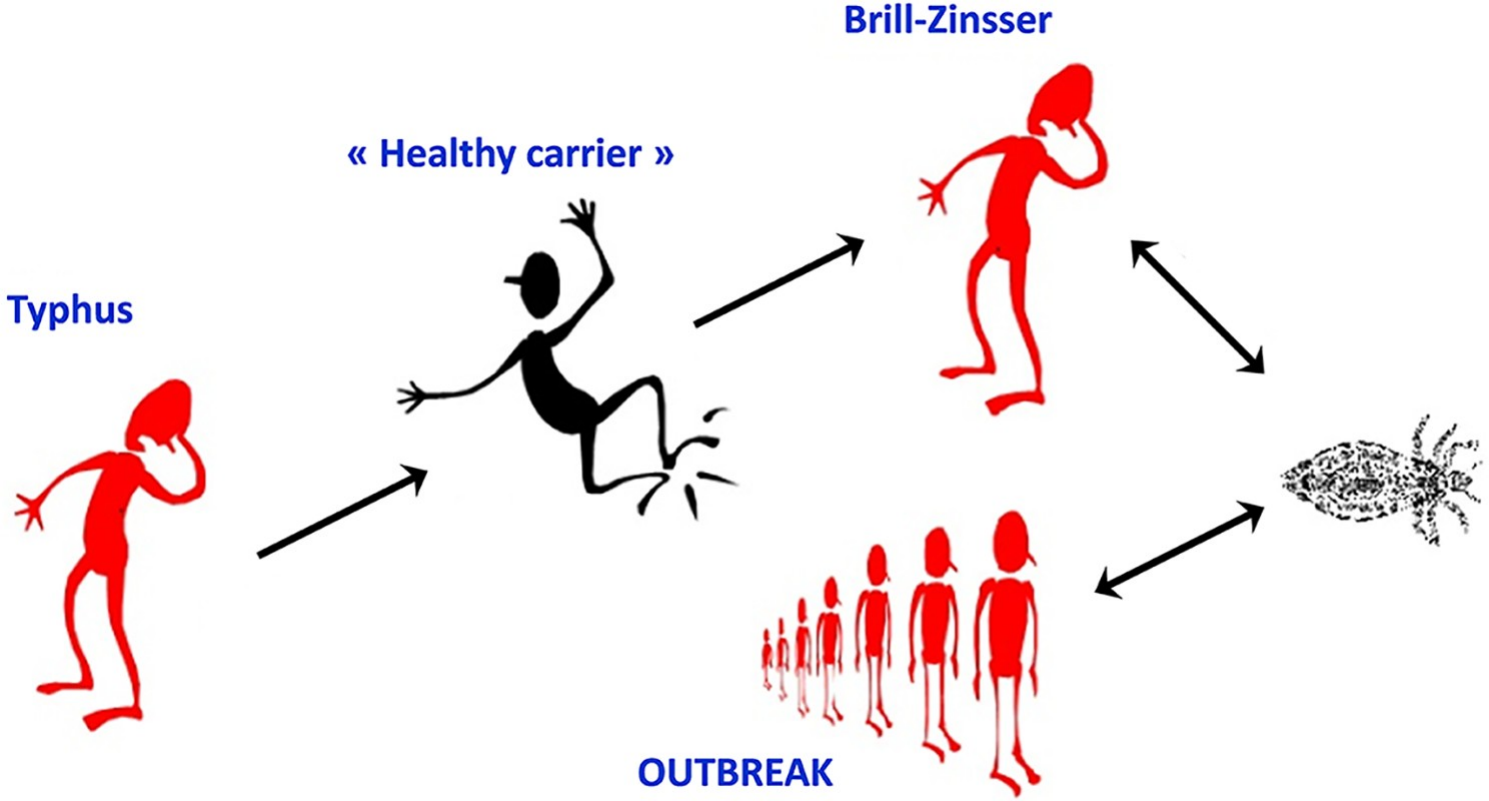
Body lice as the cause of typhus reemergence. *R*. *prowazekii* is classified as a category B bioterrorism agent. It is stable in dried louse feces and can be transmitted through aerosols. Detection of the pathogen in body lice is crucial for monitoring the transmission risk to humans. However, Brill–Zinsser disease, a relapsing form of epidemic typhus that may develop as sporadic cases up to 40 years after the initial acute infection, is unrelated to louse infestation but to stress or a waning immune system that initiates the reactivation of an earlier and latent infection. Patients developing Brill–Zinsser disease may, in turn, be the source of new outbreaks when conditions facilitate louse infestation and transmission [155]. The mechanism of *R*. *prowazekii* latency has not been established. Brill–Zinsser disease should be considered as a possible diagnosis for acute fever in any patient who has lived in an area where epidemic typhus was endemic [156].

Rickettsiae are strictly intracellular bacteria that are vectored by various arthropods. To date, 18 species are recognized human pathogens, 12 of which cause CNS R (Fig 1). To these pathogenic *Rickettsia* species may be added *O*. *tsutsugamushi* and *O*. *chuto* that are phylogenetically closely related and are still often referred to as rickettsiae. Rickettsioses are zoonotic diseases that distributed worldwide (Fig 2) in foci of endemicity (Table 1), with sporadic and often seasonal outbreaks [11]. However, *R*. *prowazekii* has the potential to reemerge as outbreaks in human populations living in close proximity and impoverished conditions [12] (Fig 3).

**Table 1:** Geographical distribution and arthropod vectors of *Rickettsia* and *Orientia* species causing CNS infections.

Species (type strain)	Disease	Vector	Distribution	Genome sequence accession number*
*R*. *africae* (ESF-5)	ATBF	*Amblyomma* and *Rhipicephalus* ticks	Sub-Saharan Africa, West Indies	NC_012633
*R*. *akari* (Hartford)	Rickettsialpox	*L*. *sanguineus* mites	North America, Asia, Europe	NC_009881
*R*. *australis* (Cutlack)	QTT	*Ixodes* ticks	Australia	NC_017058
*R*. *conorii* (Malish 7)	MSF	*Rhipicephalus sanguineus* ticks	Europe, North Africa, Asia	NC_003103
*Rickettsia felis* (Marseille-URRWFXCal_2_)	Flea-borne rickettsiosis	Mostly *C*. *felis*	Worldwide	NC_007109.1
*Rickettsia helvetica* (C9P9)	Unnamed rickettsiosis	*Ixodes* sp.	Europe, Asia, North Africa	NZ_CM001467
*Rickettsia japonica* (YH)	Japanese/Oriental spotted fever	*Haemaphysalis* ticks	Asia	NC_016050
*Rickettsia raoultii* (Khabarovsk)	TIBOLA	*Dermacentor* and *Ixodes* ticks	Europe	NZ_CP010969
*Rickettsia rickettsii* (Sheila Smith)	RMSF	Mostly *Dermacentor* ticks	North and Central America	NC_009882
*Rickettsia slovaca* (13-B)	TIBOLA	*Dermacentor* ticks	Europe, Asia	NC_016639
*R*. *prowazekii* (Madrid E)	Epidemic typhus	*Pediculus humanus humanus* lice	Africa, Russia, South America	NC_000963
*R*. *typhi* (Wilmington)	Murine/endemic typhus	*X*. *cheopis* fleas	Worldwide	NC_006142
*O*. *tsutsugamushi* (Ikeda)	Scrub typhus	*Leptotrombidium* mites	Asia	NC_010793

*All genomes were imported from GenBank (http://www.ncbi.nlm.nih.gov/genome/)

**Abbreviations**: ATBF, African tick bite fever; CNS, central nervous system; MSF, Mediterranean spotted fever; QTT, Queensland tick typhus; RMSF, Rocky Mountain spotted fever; TIBOLA, tick-borne lymphadenopathy

## Clinical manifestations of rickettsial infections

Spotted fever rickettsioses typically present with a triad made of fever, inoculation eschar(s), and generalized cutaneous rash. However, infected patients may develop other clinical pictures, including nonspecific flu-like symptoms, isolated fever, myalgia, cough, generalized lymphadenopathy, abdominal pain, and a variety of neurological symptoms that are described next.

Most commonly, patients recover without sequellae. However, the clinical presentation of rickettsial diseases may vary from mild to very severe, with the case-fatality rate for highly virulent rickettsiae ranging from 2% to 30% [13]. The severity of rickettsial disease has been associated with differences in pathogen virulence and host-related factors (e.g., age, delayed diagnosis, hepatic and renal dysfunction, CNS and lung involvement) [14]. Regarding species-specific variations in virulence, studies have suggested that these differences may be linked to the degree of genomic degradation. In a comparison of 4 species exhibiting distinct virulence profiles, the most virulent *Rickettsia* sp. exhibited the most drastically reduced and degraded genomes compared with closely related species of milder pathogenesis [15].

## Mechanisms of bacterial entry into the CNS

**Fig 4:**
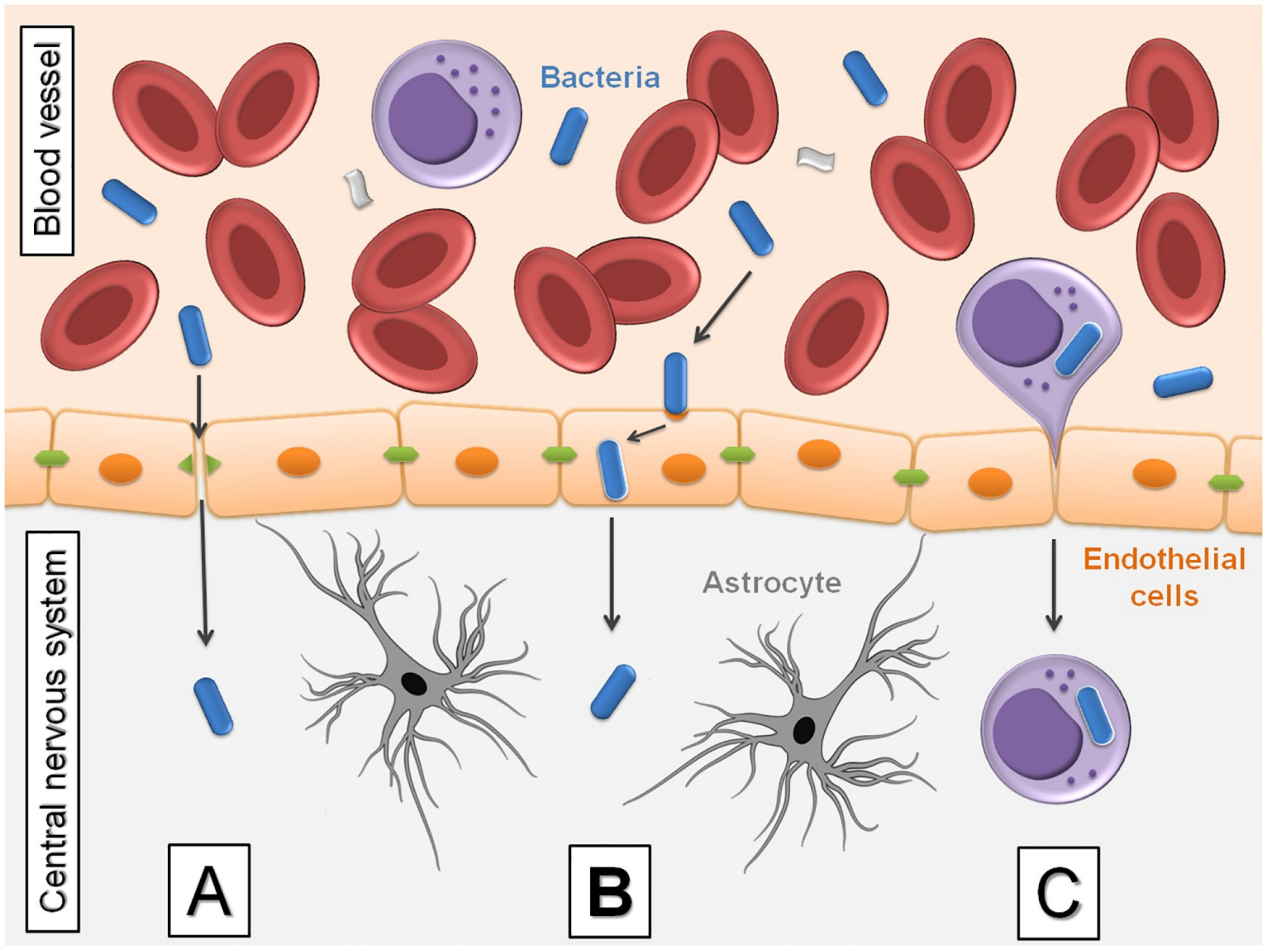
Mechanisms of bacterial penetration through the blood–brain barrier. (A) Intercellular or paracellular, described in extracellular pathogens. The physical barrier formed by endothelial tight junctions is disturbed by bacterial penetration. (B) Transcellular, passing through cells, e.g., direct invasion of endothelial cells. In this scenario, blood-borne bacteria directly invade CNS endothelial cells. (C) By leukocytes, within infected macrophages, named “Trojan horse” mechanism. Infected leukocytes adhere to endothelial cells, allowing the spread of bacteria or, alternatively, leukocytes can transmigrate and deliver bacteria to the CNS parenchyma. CNS, central nervous system.

The brain–blood barrier (BBB) at the level of brain microvessel endothelium is the major site of blood–CNS exchange. The mechanisms of bacterial entry into the CNS are divided according to whether they break the integrity of BBB cells (which consist of microvascular endothelial cells of the brain, pericytes, and astrocytes) or interact with epithelial cells of the blood–choroid barrier. Mechanisms used by pathogens to enter the CNS are usually classified according to the involved cellular route [16,17]. These cellular entry routes are listed as intercellular or paracellular (passing between cells), transcellular (the intracellular mode), or leukocyte-facilitated in infected phagocytes (which is a “Trojan horse”-like mechanism) [18,19]. Rickettsial neuroinvasion occurs during the systemic phase of the disease and typically follows bacterial dissemination via the bloodstream. As other intracellular microorganisms, rickettsiae might use a transcellular approach, which is supported by the fact that bacterial replication takes place within endothelial cells. Other studies have demonstrated that bacterial pathogens (extra- and intracellular) can reach the BBB and are recognized by antigen-presenting cells through binding to Toll-like receptors (Fig 4).

They induce the activation of nuclear factor-kappa B- or mitogen-activated protein kinase pathways and subsequently up-regulate leukocyte populations and express numerous proteins involved in inflammation and the immune response [16,18]. However, the precise molecular mechanisms of rickettsial entry into the CNS are not yet described.

## Treatment of rickettsioses

The most efficient antibiotics to cure rickettsioses are tetracycline derivatives, primarily doxycycline. The daily dose, 200 mg, is usually administered orally and is sufficient to cure rickettsioses. Treatment duration is 7 days or until 2 days after apyrexia is obtained [20,21]. In severe forms, intravenous doxycycline may be used. Alternatively, chloramphenicol and macrolides (azithromycin, clarithromycin, and roxytromycin) can be prescribed for treating rickettsial diseases [22,23]. As a consequence, empirical therapy for CNS infections most often does not include antibiotics active on rickettsiae.

## Neuropathology in the framework of rickettsial diseases

### Spotted fever group rickettsiae

In spotted fever group (SFG) rickettsioses, encephalitis is underdiagnosed due to underrecognition and low sensitivity of serologies at the time of early symptoms [24]. Therefore, it is difficult for clinicians to recognize the clinical, biological, and neuroimaging features of this type of encephalitis [25].

### Rocky Mountain spotted fever

Rocky Mountain spotted fever (RMSF), caused by *R*. *rickettsii*, frequently presents with neurological symptoms [26,27]. The CNS notably appears to be one of the major systems involved during the latest stages of RMSF pathogenesis [28].

The onset of symptoms is usually abrupt, with severe headache, fever, chills, myalgias, arthralgias, and prostration. Neurological symptoms are frequently a prominent feature. Headache, restlessness, insomnia, and back stiffness are common. Delirium or coma alternating with restlessness is present during the peak of fever [26,27,29–31]. Encephalitis, which may be fatal, is a frequent manifestation in case of delayed diagnosis and treatment [32]. In addition, several authors have described severe sequelae in patients who survived *R*. *rickettsii* infection, including hemiplegia, deafness, visual disturbance, slurred speech, mental confusion, and cranial neuropathies that may persist for a few weeks following recovery [33–37].

Histological studies have highlighted that the pathologic changes in RMSF are most severe in the skin, but the heart, lungs, and CNS also are involved. The brain is edematous, and minute petechial hemorrhages are present. The characteristic microscopic lesions are small round nodules composed of elongated microglia, lymphocytes, and endothelial cells [29,38,39]. These are scattered diffusely through the brain in close relation to small vessels. Vessels in the center of the lesions show severe degeneration. Endothelial cells are swollen, and the lumen may be occluded. Areas of focal necrosis are common as the result of thrombosis of small arteries. Some degree of perivascular infiltration without the presence of nodules may be seen in both the meninges and brain parenchyma [29].

To the best of our knowledge, there is just one report of experimental in vitro rickettsial infection of neuronal cells. Joshi and Kovacs [40] demonstrated that neurons are efficiently infected by *R*. *rickettsii* and that the infection causes significant neuronal apoptotic cell death. A double-immunofluorescent staining technique was employed to identify infected neurons, using anti-*R*. *rickettsii* serum and a neuron-specific (antineuronal *β*-III isoform of tubulin [anti-TuJ1]) antibody. The extent of apoptotic cell death of immunocytochemically identified neurons was determined by terminal deoxynucleotidyl transferase-mediated digoxigenin-dUTP nick-end labelling (TUNEL) assay. Primary cultures of rat cerebellar granular neurons were infected (approximately 65% of neurons) during a 6-hour exposure to *R*. *rickettsii*, and the infection induced widespread neuronal degeneration. To verify that the observed neuronal degeneration was *R*. *rickettsii*-induced, primary cultures of rat cerebellar granular neuron cultures were exposed to *R*. *rickettsii* in the presence of tetracycline. The authors also included *R*. *rickettsii* infection of endothelial cells as a control for infection and infection-induced apoptotic cell death [40].

The CNS is also a crucial target in other rickettsial diseases [41,42]. It has been demonstrated by a specific immunohistochemistry technique, during the development of mouse-model studies, that rickettsiae reach brain tissues when injected intravenously [43]. Such studies demonstrate that rickettsiae are able to cross the blood–brain barrier to reach brain tissues. The other rickettsial infections that may affect the nervous system exhibit pathological and clinical changes similar to those of RMSF [29]. In rickettsial meningitis, the cerebrospinal fluid shows pleocytosis with lymphocyte predominance, hypoglycorrhachia, and hyperproteinorrhachia.

### Mediterranean spotted fever

Mediterranean spotted fever (MSF), also known as Boutonneuse fever and Marseilles fever, is caused by *R*. *conorii*. Although usually a benign and self-limiting exanthematous febrile illness [44–47], MSF has a fatality rate of 2% to 7% among hospitalized patients [48]. In large MSF case series from France and Spain, patients with severe and fatal forms of the disease developed complications, including acute renal failure, thrombocytopenia, myocarditis, pneumonitis, gastric hemorrhage, shock, and multiple organ failure [44–47,49]. Furthermore, a few reports in the literature have described CNS involvement in the course of MSF, presenting as meningitis [50,51], encephalitis [45,47,49,50,52,53], meningoencephalitis [47,54–56], or myelitis [50]. Among the patients who survived these severe forms, only 2 did not suffer sequelae. Sequelae were severe, despite an appropriate doxycycline treatment of 200 mg every 12 hours [54,57].

### Japanese spotted fever

Japanese spotted fever (JSF), caused by *R*. *japonica*, was first reported in 1984 [58]. This rickettsial disease is characterized by high fever, rash, and inoculation eschar at the tick bite site [58]. JSF is clinically similar to scrub typhus, caused by *O*. *tsutsugamushi*, but often with a more severe course [58]. CNS involvement is rare, but cases complicated by pneumonia or meningoencephalitis have been reported [59–61]. There have been only 5 JSF reported cases with CNS involvement [62–64]. The first fatal case was reported by Kodama and colleagues in 2002 [65]. All patients exhibited fever and mental disturbance, including 3 patients with nuchal rigidity and 3 patients with seizures. All patients had aseptic meningitis [66].

### Tick-borne lymphadenopathy

*R*. *slovaca* and *R*. *raoultii* cause a rickettsiosis that has successively been named tick-borne lymphadenopathy (TIBOLA) [67–69], *Dermacentor*-borne necrosis erythema and lymphadenopathy (DEBONEL) [70–73], and then scalp eschar and neck lymphadenopathy after tick bite (SENLAT) [74]. The first *R*. *slovaca* isolate was cultivated from a *D*. *marginatus* tick collected in central Slovakia and was initially considered nonpathogenic [67,75,76]. However, in 1997, *R*. *slovaca* was identified as the cause of a clinical entity combining scalp eschar and neck lymphadenopathy after a tick bite [76]. After MSF, TIBOLA is nowadays the most prevalent tick-borne rickettsiosis in Europe [77].

This syndrome is defined by the association of a tick bite and an inoculation eschar to the scalp, surrounded by a circular erythema, and painful regional lymphadenopathies [78]. The enlarged lymph nodes are located in the draining region of the tick bite, characteristically in the occipital region and/or behind the sternocleidomastoidal muscle. Although rare, the most frequent general symptoms are fever, nuchal lymphadenopathy, fatigue, dizziness, headache, sweat, myalgia, arthralgia, and loss of appetite [68,79]. Symptoms suggestive of acute encephalitis with febrile relapses and a persistence of neurasthenic disorders have also been reported [68,80].

Although *R*. *slovaca* was the first demonstrated agent of TIBOLA [76] and was isolated from a patient [81], other *Rickettsia* species were suspected to be involved in the disease in Spanish reports [71,82]. In 2008, *R*. *raoultii* DNA was detected in a tick taken from the scalp of a patient with TIBOLA [83]. In 2009, Parola and colleagues reported 6 more cases of *R*. *raoultii* infection [84]. To date, *R*. *raoultii* has been described as causing a milder form of TIBOLA than *R*. *slovaca* [84].

### African tick bite fever

African tick bite fever (ATBF), caused by *R*. *africae*, is the most common rickettsiosis in travelers to sub-Saharan Africa and also the most important rickettsiosis worldwide in terms of numbers of cases per year [85–87]. The disease is most commonly mild and self-limiting without any sequellae. However, in 2006, Jensenius and colleagues reported 6 patients with evidence of long-lasting subacute neuropathy following ATBF contracted during a safari to southern Africa [88]. Of these, 3 developed radiating pain, paresthesia, and/or motor weakness of the extremities; 2 had hemifacial pain and paresthesia; and 1 developed unilateral sensorineural hearing loss.

### *R*. *helvetica* infection

*R*. *helvetica* was first isolated from *Ixodes ricinus* ticks in Switzerland in 1979 [89], but its description as a distinct species of the SFG was confirmed only in 1993 [90]. Initially, the organism was considered as nonpathogenic. However, several patients with a mild and self-limiting disease associating fever, headache, and myalgia had serological evidence of *R*. *helvetica* infection [91]. Human cases have been reported in several European countries, e.g., Sweden [92] (proven by PCR and DNA sequencing), France [91], Switzerland [93], and Italy [94] (proven by serological findings). However, a more severe clinical disease has been reported and may be associated with severe symptoms such as CNS disorders [95,96] (verified by PCR in the cerebrospinal fluid, together with serologic evidence). *R*. *helvetica* was even cultured from a patient with subacute meningitis [97].

### Typhus group rickettsiae

#### Epidemic typhus

*R*. *prowazekii*, the agent of epidemic typhus, causes frequent neurological manifestations. These include an agitated delirium that, when untreated, may progress to death. A severe headache is almost always present. Neurological complications include seizures, confusion, and coma. In severe cases, patients develop meningoencephalitis, with meningism, tinnitus, and hyperacusis, followed by deafness, dysphoria, and agitation. In Brill–Zinsser disease, the recurrent form of epidemic typhus that may occur months to years after the initial infection, symptoms are similar to the primary infection, although generally milder [29,98].

#### Murine typhus

*R*. *typhi*, the causal agent of murine or endemic typhus, is transmitted to humans by the rat flea *Xenopsylla cheopis*. It is most often a mild illness, but severe forms have been reported in refugee camps, and a fatal case in the United Kingdom was infected in Spain [99]. There is a considerable variability in the frequency of neurological manifestations of murine typhus, ranging from 2% to 20% of cases [100,101]. Headache is common, but meningitis and encephalitis are occasionally reported [102]. Mental confusion, seizures, stupor, and ataxia may occur infrequently [101,103–105]. In some cases, systematic signs and symptoms may be minimal, and neurological manifestations may be the primary clinical manifestations of *R*. *typhi* infection. No neurologic sequellae have been reported in the past [29,42]. However, recently, an abducens nerve palsy and meningitis induced by *R*. *typhi* has been described [106].

### Transitional group rickettsiae

#### Rickettsialpox

Rickettsialpox was first described in 1946 in New York City, New York [107]. The etiological agent is *R*. *akari*, transmitted by the mite *Liponyssoides sanguineus* [108]. Rickettsialpox is still occurring in the state of New York [109,110], and European cases of *R*. *akari* infection were reported in Ukraine [111], Croatia [112], and Northern Europe [113]. Clinical signs of *R*. *akari* infection develop about 7 to 10 days after an infected bite. At the mite inoculation site, a vesicle appears, which dries up, leaving a scar. High fever (≤39.4 °C) occurs suddenly, along with severe headache and myalgias (especially in the back). A generalized papular rash develops, which can also be vesicular, resembling chickenpox. Other signs of the disease include regional lymphadenopathy, nausea, and neurological symptoms such as meningitis, photophobia, dizziness, eye movement, and neck stiffness. The disease is self-limiting, usually without complications, but myalgias and headache may persist for 2 more weeks after the rash [114].

#### Queensland tick typhus

*R*. *australis*, the causative agent of Queensland tick typhus (QTT), is transmitted by certain *Ixodes* spp. ticks, which are predominantly present along the eastern coast of Australia [115]. Originally described as a mild, self-limiting illness, recent case reports demonstrated the occurrence of more severe forms with complications. *R*. *australis* shares many clinical features with rickettsialpox. Acute infections often begin with high-grade fever, headache, myalgias, and a maculopapular rash after a short incubation period averaging 5 days [116,117]. Infection has the potential to progress to severe sepsis with multiorgan failure, requiring prolonged hospitalization and admission to intensive care [118]. Since *R*. *australis* was first isolated in 1946, there has been one reported fatal QTT case [119]. Less common manifestations of QTT include splenomegaly, abdominal pain, renal failure, dry cough, and conjunctivitis [120]. Some reports documented confusion, seizures, and hallucinations as features of the disease [116].

#### Flea-borne spotted fever

Flea-borne spotted fever is caused by *R*. *felis*. It is transmitted to humans by fleas, in particular *Ctenocephalides felis* fleas. Patients often present with elevated fever, headache, and myalgia. A cutaneous rash is inconstant. Other manifestations may include abdominal pain, nausea, vomiting, cough, eschar, and photophobia. Rare cases of neurological involvement have been reported, notably, hearing loss and subacute meningitis [121,122].

### Scrub typhus

The genus *Orientia* contains two known species, *O*. *tsutsugamushi* and *O*. *chuto* [123], that cause scrub typhus, which is still regarded by some experts as a rickettsiosis, and are closely related to *Rickettsia* species [124,125]. Scrub typhus is a common cause of febrile illness in Asia. Fever and headache are the most common features of scrub typhus [126]. During World War 2, scrub typhus was a well-recognized cause of lethal meningitis in the Asia–Pacific region. Nowadays, only scarce data exist on the clinical burden of these pathogens in patients with CNS disease, e. g., in Laos and Thailand, where meningitis and meningoencephalitis occur as severe complications [127,128]. Isolated cases of acute encephalitis, abducens nerve palsy, and acute transverse myelitis due to *O*. *tsutsugamushi* were also recently described [129,130].

### Rickettsia-related organisms

Even though we have focused our review on members of the genus *Rickettsia*, we have searched the literature for CNS infection caused by the related organisms *Ehrlichia* and *Anaplasma* species.

Neurological involvement in human monocytic ehrlichiosis, caused by *E*. *chaffeensis*, is not rare [131–139]. Severe headache and symptoms of meningeal irritation are present in as many as 10% of cases [140]. Most cases present as aseptic meningitis, but complications such as ataxia, cranial nerve paresis, meningoencephalitis, multiorgan failure, optic neuritis, seizures, and demyelinating polyneuropathy have been reported [137,138,141–143]. In addition, *E*. *canis* was detected in a child with aseptic meningitis [144].

*A*. *phagocytophilum* appears to be a rare agent of CNS infections [145], but cases of cerebral infarction or brachial plexopathy associated to *A*. *phagocytophilum* infection were reported [146,147]. In addition, *A*. *phagocytophilum*-infected neutrophils were described to enhance transmigration of *Borrelia burgdorferi* across the human blood–brain barrier in vitro [148].

## Conclusion

Through a literature review, we identified 13 rickettsioses, including scrub typhus, causing CNS infections ranging from simple headache to lethal meningoencephalitis. In addition, surviving patients may suffer incapacitating sequellae. To date, the precise mechanisms of rickettsial pathogenesis for the CNS are only partially known, but animal models have demonstrated their pro-apototic effect for neurons. However, these bacteria are constantly susceptible to tetracyclines that may efficiently be used to cure patients. The remaining challenge is to integrate rickettsioses more systematically in the differential diagnosis of CNS infections. Without this effort, the diagnosis and treatment may be delayed, increasing the risk of severe forms.

**Top five papers**

- Context: Parola P, Paddock CD, Socolovschi C, Labruna MB, Mediannikov O, Kernif T, Abdad MY, Stenos J, Bitam I, Fournier PE, and Raoult D (2013) Update on tick-borne rickettsioses around the world: a geographic approach. Clin Microbiol Rev, 26, 657–702.
- Pathogenesis: Joshi SG and Kovacs AD (2007) Rickettsia rickettsii infection causes apoptotic death of cultured cerebellar granule neurons. J Med Microbiol, 56, 138–141.
- Epidemiology: Sexton DJ and Kirkland KB (1998). Rickettsial infections and the central nervous system. Clin Infect Dis,26, 247–248.
- Diseases: Bleck TP (1999) Central nervous system involvement in Rickettsial diseases. Neurol Clin, 17, 801–81.
- Diseases: Araki M, Takatsuka K, Kawamura J, and Kanno Y (2002) Japanese spotted fever involving the central nervous system: two case reports and a literature review. J Clin Microbiol, 40, 3874–3876.

**Key learning points**

- Rickettsioses are arthropod-transmitted and zoonotic diseases with a worldwide distribution.
- Rickettsioses represent a frequent threat for travelers to rural areas but are often misdiagnosed.
- Eleven of the currently diagnosed rickettsioses cause CNS infections in humans.
- Rickettsial CNS infections range from meningitis to lethal encephalomyelitis.
